# Distinct Prognostic Role of Serum Uric Acid Levels for Predicting All-Cause Mortality Among Chinese Adults Aged 45~75 Years With and Without Diabetes

**DOI:** 10.3389/fendo.2021.782230

**Published:** 2021-11-16

**Authors:** Bowen Zhu, Jian Zhang, Nana Song, Yiqin Shi, Yi Fang, Xiaoqiang Ding, Yang Li

**Affiliations:** ^1^ Department of Nephrology, Zhongshan Hospital, Fudan University, Shanghai, China; ^2^ Shanghai Medical Center of Kidney, Zhongshan Hospital, Fudan University, Shanghai, China; ^3^ Shanghai Key Laboratory of Kidney and Blood Purification, Zhongshan Hospital, Fudan University, Shanghai, China

**Keywords:** serum uric acid, diabetes, all-cause mortality, epidemiology, China Health and Nutrition Survey

## Abstract

**Introduction:**

The current study sought to explore the effect of baseline serum uric acid (SUA) on the risk of all-cause mortality among Chinese adults aged 45~75 years and to determine its interaction relationship with diabetes.

**Methods:**

The study was designed as a community-based cohort of 4467 adults aged between 45~75 years included in a 6-years follow-up period from 2009 to 2015 years by the China Health and Nutrition Survey (CHNS). Baseline SUA levels were grouped into quartiles and its association on all-cause mortality was explored using multivariate Cox proportional hazards models. Stratified analyses were performed to explore the associations of SUA quartiles with all-cause mortality among diabetic and non-diabetic individuals.

**Results:**

A total of 141 deaths (5.3 per 1000 person-years) were recorded During a follow-up of 26431 person-years. Out of the 141 deaths, 28 deaths (10.1 per 1000 person-years) were reported in the diabetic groups and 113 deaths (4.8 per 1000 person-years) were recorded in the non-diabetic group. An increased risk of all-cause mortality was observed for participants in the first and fourth quartiles compared with the second SUA quartile, (Q1 SUA: aHR=2.1, 95% CI 1.1~4.1; Q4 SUA: aHR=2.1, 95% CI 1.1~4.0). Stratification of participants by diabetes status showed a U-shaped association for non-diabetic individuals. Whereas, declined eGFR, rather than SUA, was an independent risk factor for all-cause mortality in diabetic individuals (aHR=0.7, 95% CI 0.6~1.0).

**Conclusion:**

Our study proved that the prognostic role of SUA for predicting all-cause death might be regulated by diabetes. Both low and high SUA levels were associated with increased mortality, supporting a U-shaped association only in non-diabetic individuals. Whereas, renal dysfunction rather than SUA was an independent risk factor for all-cause mortality. Further studies should be conducted to determine the SUA levels at which intervention should be conducted and explore target follow-up strategies to prevent progression leading to poor prognosis.

## Introduction

Prevalence of hyperuricemia (HUA) is more than 20% of the general population and presents a rapid increase globally (1). In China, the disease burden of HUA has risen significantly over the recent decades, from approximately 8.5% in 2001 to approximately 18.4% in 2017 (2). Clinical trials report that elevated serum uric acid (SUA) is a strong predictor of poor outcomes in cardiovascular death, acute ischemic stroke, and all-cause death (3–6). Previous studies report that low and high UA levels are correlated with increased mortality with approximately 1.5~5.0 times higher risk. Notably, 1 mg/dL UA levels increase is related to 3 times higher risk, and a U-shaped association occurs between SUA levels and adverse health outcomes (7, 8).

Noteworthy, the risk ratio varies with types of diseases in HUA patients including hypertension, diabetes, chronic kidney disease (CKD) and metabolic syndromes (9–14). A previous study reported that HUA patients have a higher all-cause mortality rate compared to the general population, which is mainly attributed to cardiovascular diseases (CVD), metabolic syndromes and diabetes (15).

Studies should explore whether SUA and diabetes independently or jointly affect prognosis as HUA has a high prevalence and accounts for 13.0%~35.0% of patients with diabetes and owing to the disparity of UA levels in different associated diseases (16–18). Studies on the association between SUA level and mortality in diabetic individuals report conflicting findings. Lamacchia et al. report that SUA was not linearly associated with all-cause mortality in diabetic patients and a higher risk of mortality was observed for the first and third SUA tertiles (HR: 1.34 and 1.61) (19). Another 9-year cohort study reported significantly interactive effect of uric acid with diabetes (RR=1.26) on the risk of all-cause mortality, whereas the findings showed that the effects of uric acid were not significant (20). However, most of the previous studies did not conduct appropriate comparisons (21, 22), and recruited subjects from hospitals (23), thus limiting the ability to fully explore whether SUA independently contributes or acts synergistically with renal function associated with diabetes. Notably, studies have not explored the potential role of SUA on quality of life in the Chinese population with diabetes.

Therefore, we designed a 6-year cohort study based on the China Health and Nutrition Survey (CHNS) to explore the effect of baseline serum uric acid (SUA) on the all-cause mortality among Chinese adults aged 45~75 years and determine its interaction relationship with diabetes was determined.

## Materials and Methods

### Study Cohorts

The CHNS is an ongoing nationwide prospective cohort study that comprised the Chinese population. The study sought to explore the effects of the health, nutrition, and family planning policies and programs implemented by national and local governments. CHNS was conducted in 1989, and it was subsequently performed in 1991, 1993, 1997, 2000, 2004, 2006, 2009, 2011 and 2015. Details on the design and data collection of CHNS have been reported previously (24). Data on demographic, economic circumstances, diet, behaviors, and health were collected from each household member for all CHNS waves. Written informed consent was obtained from all participants. CHNS was approved by the Institutional Review Board at the University of North Carolina at Chapel Hill and local IRB (institutional review board or ethics committee). Blood samples were collected in 2009. A total of 9546 participants were included in the 2009 wave of CHNS and data on biomarkers were obtained from these subjects. Moreover, death-related information was obtained for the 2009, 2011 and 2015 cohorts.

Considering participants aged above 75 years may exert the effect of age on aging-related variables and no cases of deaths under the age of 45 years were observed due to CHNS being a community-based dataset. Participants aged 45 to 75 years were excluded from the study. A total of 378 (7.8%) participants were excluded as they had less than two visits during the follow-up duration. Demographic and behavioral characteristics of participants included in the final analysis (n=4467) were compared with that of excluded participants (n=378) (**Supplementary Table 1**).

### Data Collection

A standardized structured questionnaire was administered by trained health staff to collect socio-demographic variables (in 2009) including age, gender, educational attainment, urban-rural residence, history of diseases (e.g. hypertension, diabetes), smoking habits, drinking status, tea intake, coffee intake, total protein intake and physical activity level. Physical examinations of waist circumference, hip circumference, height, weight and blood pressures (BP) were performed by trained clinical staff (24). Biomarkers were obtained in 2009. All individuals maintained a regular pattern of life for at least three days before blood sample collection and were required to be collected 12 ml blood (in three 4 ml tubes) on empty stomach. The details were seen on the website: http://www.cpc.unc.edu/projects/china/data/datasets/biomarker-data. Biomarker data collected in CHNS 2009 involves the release of 26 fasting blood measures on individuals aged 7 and older (25). Totle cholesterol (TC) was measured using the Picric acid method (Hitachi 7600, Kyowa, Japan). Low-density lipoprotein cholesterol (LDL) was measured using the Enzymatic method (Hitachi 7600, Kyowa, Japan). Triglyceride (TG) was measured using the GPO-PAP (Hitachi 7600, Kyowa, Japan). Creatinine was measured by certified technicians at each field center using the CHOD-PAP (Hitachi 7600, Randox, UK).

The individuals were categorized as groups, namely, for residence, into urban and rural; for educational attainment, into 0, 6 years or less, 6–8 years, 9–11 years, and 12 years or higher; for smoking status into non-smokers, ex-smokers and current smokers; for alcohol status, into drinker and non-drinker. Individual dietary intake for 3 consecutive days was determined for every household member. This step has been achieved by asking individuals each day to report all food consumed away from home on a 24-hour recall basis, and the same daily interview has been used to collect at-home individual consumption. BMI was calculated as weight (in kilograms) divided by the square of height (in meters) and categorized into four levels including: lean (< 18.5 kg/m^2^), normal (18.5~23.9 kg/m^2^) and overweight (24.0~27.9 kg/m^2^) and obese (≥28 kg/m^2^). Waist-to-hip ratio (WHR) was calculated as waist circumference (cm)/height (cm). The cutoffs for the WHR were identified as 0.9 for men and 0.85 for women, using the World Health Organization (WHO) guidelines (26). Systolic blood pressure (BP) and diastolic blood pressure were expressed as the mean of three measurements. Hypertension was defined as systolic blood pressure (BP) ≥140 mmHg or diastolic BP ≥90 mmHg or self-reported (27). Diabetes mellitus was defined as HbA1c ≥6.5% or self-reported or having diabetes treatment records. Dyslipidemia was defined as total cholesterol level at 5.2 mmol/L or higher, LDL cholesterol level at 3.4 mmol/L or higher, or triglycerides level at 1.7 mmol/L or higher (28). Estimated glomerular filtration rate (eGFR) was calculated using the chronic kidney disease epidemiology collaboration (CKD-EPI) (29). The Framingham risk score (FRS) is based on an algorithm derived from a 10-year predicted risk of CVD estimate comprised of age, sex, total cholesterol, HDL cholesterol, smoking history, blood pressure, and diabetes (30). 10-year CVD FRS was classified as low (<10%), intermediate (10-20%), or high risk (>20%).

The quality of the data collection was controlled. The design and implementation of the survey detailed the protocol for training the field staff for data collection and office staff for data entry and how to properly check and clean the data. These procedures have become an established part of work in China after regional staff complete extended trips to the U.S. for training (24).

### Exposure

In the 2009 wave of CHNS, 12 ml blood was collected from participants on an empty stomach. Data were deposited on the website: https://www.cpc.unc.edu/projects/china. SUA was selected as the exposure variable. SUA was determined by certified technicians at each field center by the enzymatic colorimetric method (Hitachi 7600, Randox, UK), following the standard procedures. SUA levels were categorized into quartiles (<4.7, 4.7~5.5, 5.6~6.9, >6.9 mg/dL for men and <3.7, 3.7~4.4, 4.5~5.4, >5.4 mg/dL for women).

### Analysis of All-Cause Mortality

The variable wave in CHNS represents the “year” of study. The date of death was reported in each wave (wave=2009, 2011 and 2015). Follow-up duration was determined as the time from baseline (survey date in 2009) till the date of death recorded during CHNS or the censoring time. A total of 389 participants were excluded from the study for having less than two visits during the follow-up. In sensitivity analysis, if the event of all-cause death was recorded on the same day as the baseline, the follow-up duration was recorded as 0.5 years or deleted.

### Statistical Analysis

Data were presented as mean ± standard deviation (SD) and median with interquartile range (IQR) for continuous variables or N (%) for categorical variables. Data on demographics, physical examinations, and anthropometric indexes were compared between SUA quartiles using the chi-square test and Fisher’s exact test for categorical variables. Variance analysis or Wilcoxon rank-sum test were performed for continuous variables. All-cause mortality for SUA quartiles was compared using the Kaplan-Meier (K-M) curve, and the significance was calculated using the log-rank test. Hazard ratios for mortality outcomes were estimated using Cox proportional hazards regression analysis. The second quartile of SUA was used as a reference to explore the statistical power. Multivariable Cox proportional hazards regression was adjusted for: age, gender, BMI, WHR, hypertension, smoking status, drinking status and total protein intake to determine whether each level of SUA quartiles was independently associated with all-cause mortality. Marginal structure model (MSM) was used for inverse-probability weighting analysis to determine the robustness of the findings. Participants who died in the same year were excluded or counted as 0.5 person-year during sensitivity analysis. Prespecified subgroup analyses were conducted based on age, gender, WHR, hypertension, dyslipidemia, CKD, and FRS in secondary analyses. Demographic characteristics of participants in the analytic sample were compared in case the biomarker for SUA was missing and the sample was excluded to explore potential selection bias on study results. The results are presented as hazard ratios (HR) or risk ratios (RR) with 95% confidence intervals (95% CI). P < 0.05 (two-sided) was considered statistically significant. Analysis was performed only on the available data. Data were analyzed using SAS version 9.3 (SAS Institute Inc).

## Result

### Characteristics of the Study Participants

The initial cohort contained 8474 participants included in this study. Of them, participants (n=3996) were excluded on the first round for aging <45 or >75 years (n=3387), being pregnant (n=62), having average protein dietary intake for three consecutive days>110 g/day (n=371) (31) and having a history of myocardial infarction (MI) or apoplexy (n=176). Besides, 389 participants were further excluded for having less than two visits during the follow-up. In total, 4467 individuals were eligible for the formal analysis (**Supplementary Figure 1**). Demographic and behavioral characteristics of participants included in the final analysis (n=4467) and excluded samples (n=389) were compared. The findings showed that most characteristics were showed no significant difference between the two groups (**Supplementary Table 1**).

The mean age of participants was 57.7 ± 8.3 years, 45.3% of the participants were male and the mean follow-up duration was 5.9 ± 0.6 years. Notably, 475 diabetic and 3992 non-diabetic participants are presented in the study. Baseline characteristics of study patients across SUA quartiles are reported in **Table 1**. Analysis of diabetic individuals showed that individuals within the highest SUA level were more likely to be men, with the higher proportions of overweight, hypertension, dyslipidemia, CVD risk, smoking, alcohol drinking compared with the other three SUA quartiles. Moreover, non-diabetic individuals with the highest SUA level were more likely to be men and the elderly, with the higher proportions of senior-school education, urban residence, obese WHR, overweight, hypertension, dyslipidemia, smoking, drinking, and total protein intake were all higher compared with the other three SUA quartiles.

**Table 1:** Baseline characteristics of the participants and SUA quartiles among individuals with and without diabetes in CHNS Cohort.

	Diabetes (n = 475)					Non-diabetes (n = 3992)				
	Q1 (Male: <4.7; Female: <3.7)	Q2 (Male: 4.7~5.5; Female: 3.7~4.4)	Q3 (Male: 5.6~6.9; Female: 4.5~5.4)	Q4 (Male:> 6.9;Female:>5.4)	*P*-value*	Q1 (Male: <4.7; Female: <3.7)	Q2 (Male: 4.7~5.5; Female: 3.7~4.4)	Q3 (Male: 5.6~6.9; Female: 4.5~5.4)	Q4 (Male:> 6.9; Female:>5.4)	*P*-value*
Participants (n)	94	108	117	156		1016	1018	994	964	
SUA (mg/dL)	3.5 (0.5)	4.5 (0.3)	5.5 (0.3)	7.6 (2.0)	<0.001	3.4 (0.5)	4.5 (0.3)	5.5 (0.3)	7.4 (1.7)	<0.001
Age (years)	60 (8.8)	59 (8.4)	60.5 (8.5)	60.2 (8.9)	0.607	56.1 (8.2)	57.6 (8.2)	57.9 (8.1)	58.2 (8.2)	<0.001
Male (%)	27 (28.7)	44 (40.7)	54 (46.2)	93 (59.6)	<0.001	175 (17.2)	398 (39.1)	528 (53.1)	705 (73.1)	<0.001
Education (years)					0.178					<0.001
0	20 (21.3)	17 (15.7)	14 (12.0)	16 (10.3)		206 (20.3)	179 (17.6)	157 (15.8)	85 (8.8)	
≤6	37 (39.4)	38 (35.2)	40 (34.2)	52 (33.3)		346 (34.1)	363 (35.7)	339 (34.2)	324 (33.6)	
7–9	25 (26.6)	30 (27.8)	39 (33.3)	45 (28.9)		279 (27.5)	295 (29.0)	290 (29.3)	310 (32.2)	
10–12	7 (7.5)	13 (12.0)	8 (6.8)	21 (13.5)		121 (11.9)	102 (10.0)	114 (11.5)	131 (13.6)	
>12	5 (5.3)	10 (9.3)	16 (13.7)	22 (14.1)		63 (6.2)	77 (7.6)	91 (9.2)	113 (11.7)	
Rural (%)	63 (67.0)	66 (61.1)	72 (61.5)	89 (57.1)	0.481	723 (71.2)	724 (71.1)	694 (69.8)	620 (64.3)	0.002
*Anthropometry parameters*										
Obese WHR	63 (71.6)	74 (70.5)	92 (82.1)	121 (78.1)	0.146	497 (50.4)	496 (50.0)	518 (53.3)	557 (58.9)	<0.001
BMI (kg/m^2^)					0.048					<0.001
Lean (<18.5)	2 (2.1)	1 (0.9)	4 (3.4)	1 (0.6)		73 (7.2)	51 (5.0)	52 (5.2)	35 (3.6)	
Normal (18.5–23.9)	38 (40.4)	41 (38.0)	37 (31.6)	38 (24.4)		606 (59.7)	598 (58.7)	518 (52.1)	424 (44.0)	
Overweight (24.0–27.9)	41 (43.6)	41 (38.0)	47 (40.2)	70 (44.9)		277 (27.3)	307 (30.2)	316 (31.8)	386 (40)	
Obesity (≥28.0)	13 (13.8)	25 (23.2)	29 (24.8)	47 (30.1)		60 (5.9)	62 (6.1)	108 (10.9)	119 (12.3)	
Hypertension	28 (31.5)	48 (47.5)	56 (52.8)	90 (60.8)	<0.001	208 (23.5)	255 (28.4)	311 (35.0)	376 (44.0)	<0.001
Dyslipidemia	69 (73.4)	78 (72.2)	94 (80.3)	140 (89.7)	<0.001	497 (48.9)	626 (61.5)	685 (68.9)	777 (80.6)	<0.001
eGFR (ml/min/l.73m^2^)	77.6 (11.9)	75.6 (14.6)	70.2 (15.5)	68.1 (18.9)	<0.001	79.9 (11.9)	75.9 (12.2)	73.4 (13.0)	70.2 (14.6)	<0.001
eGFR<60 (ml/min/l.73m^2^)	5 (5.3)	14 (13.1)	27 (23.1)	53 (34.0)	<0.001	43 (4.2)	99 (9.7)	144 (14.5)	213 (22.1)	<0.001
Framingham score (%)	15.6 (8.2)	17.1 (8.7)	19.1 (8.4)	21.6 (7.9)	<0.001	7 (5.6)	10.3 (7.5)	12.2 (7.8)	15.3 (8.3)	<0.001
Health-related behavior										
Smoking status					0.004					<0.001
Never	77 (81.9)	75 (69.4)	74 (63.8)	93 (59.6)		852 (83.9)	732 (72.0)	622 (62.6)	506 (52.5)	
Ever	1 (1.1)	8 (7.4)	4 (3.5)	14 (9.0)		10 (1.0)	33 (3.2)	34 (3.4)	50 (5.2)	
Current	16 (17.0)	25 (23.2)	38 (32.8)	49 (31.4)		154 (15.2)	252 (24.8)	337 (33.9)	408 (42.3)	
Alcohol drinker	18 (19.2)	36 (33.3)	37 (31.6)	63 (40.4)	0.007	176 (17.3)	295 (29.0)	327 (32.9)	492 (51.0)	<0.001
Total protein intake (g)	59.1 (18.6)	62.6 (18.7)	62.3 (17.1)	65.4 (20.2)	0.079	59.8 (18.8)	61.4 (18.5)	63.7 (18.3)	66.5 (19.1)	<0.001
Carbohydrate (g)	281.8 (103.5)	263.6 (98.8)	266.0 (84.1)	276.1 (100.2)	0.474	289.8 (99.7)	292.2 (95.5)	295.8 (93.4)	293.4 (94.2)	0.559
Fat (g)	70.4 (38.4)	83.6 (94.5)	72.5 (32.3)	79.3 (54.2)	0.341	67.5 (32.4)	72.3 (36.6)	73.4 (35.3)	80.0 (37.6)	<0.001
Energy (kcal)	2024.1 (678.8)	2077.9 (1032.4)	1973.1 (526.8)	2135.3 (738.2)	0.345	2012.7 (597.8)	2080.1 (590.9)	2126 (586.2)	2210.8 (616.2)	<0.001

BMI, body mass index; BP, blood pressure; eGFR, estimated glomerular filtration rate; SUA, serum uric acid; WHR, waist to hip circumference ratio.

Data are presented as No. (%), mean± SD or median (IQR);

*P values were calculated by using student t-test or Wilcoxon test for continuous variables and χ^2^ test or Fisher exact test for categorical variables.

7 participants were not available for education level; 121 participants were not available for WHR; 537 participants were not available for hypertension; 3 participants were not available for smoking status; 1 participant was not available for drinking behavior.

### Interactive Effect of SUA and Diabetes on All-Cause Mortality

A total of 141 all-cause deaths (5.3 per 1000 person-years) were recorded during a follow-up of 26431 person-years. Out of the 141 deaths, 28 deaths (10.1 per 1000 person-years) in diabetic group, whereas 113 deaths (4.8 per 1000 person-years) were reported in non-diabetic group. The findings showed that age (per 5-year increase: HR=1.4, 95%CI 1.3~1.6), male (HR=2.5, 95%CI 1.5~4.1), rural residence (HR=1.9, 95%CI 1.2~2.9), BMI (per 5 kg/m^2^ increase: HR=0.6; 95%CI 0.5~0.8), first and fourth quartile of SUA level (Q1: aHR=2.1, 95%CI 1.1~4.1, Q4: aHR=2.1, 95%CI 1.1~4.0) were associated with risk of all-cause mortality. In addition, a significant interactive effect between SUA and diabetes on all-cause mortality was observed (Q2 SUA × diabetes: *P*=0.044; Q3 SUA × diabetes: *P*=0.034, **Table 2**).

**Table 2:** Hazard ratios of risk factors for all-cause mortality among all individuals.

	Univariable		Multivariable	
	HR (95% CI)	*P* value	HR (95% CI)	*P* value
Age, per 5 y increase	1.6 (1.4~1.8)	<.001	1.4 (1.3~1.6)	<.001
Male	2.3 (1.6~3.2)	<.001	2.5 (1.5~4.1)	<.001
Rural	1.6 (1.1~2.4)	0.017	1.9 (1.2~2.9)	0.004
Obese WHR	1.1 (0.8~1.6)	0.453	1.3 (0.9~1.9)	0.196
BMI, per 5-unit increase (kg/m^2^)	0.6 (0.5~0.8)	<.001	0.6 (0.5~0.8)	0.002
Hypertension	1.7 (1.2~2.4)	0.003	1.3 (0.9~1.8)	0.230
Diabetes	2.2 (1.4~3.3)	<.001	0.4 (0.1~2.7)	0.324
Dyslipidemia	0.8 (0.6~1.2)	0.286	0.8 (0.5~1.2)	0.242
Serum uric acid (mg/dL)				
Q1 (Male: <4.7; Female: <3.7)	1.1 (0.6~2)	0.811	2.1 (1.1~4.1)	0.031
Q2 (Male: 4.7~5.5; Female: 3.7~4.4)	Ref (1.00)		Ref (1.00)	
Q3 (Male: 5.6~6.9; Female: 4.5~5.4)	1.6 (0.9~2.8)	0.077	1.4 (0.7~2.7)	0.290
Q4 (Male:> 6.9; Female: >5.4)	2.5 (1.5~4.1)	<.001	2.1 (1.1~4.0)	0.018
Smoking status				
Never	Ref (1.00)		Ref (1.00)	
Ever	3.3 (1.8~6.1)	<.001	1.2 (0.6~2.6)	0.625
Current	1.8 (1.3~2.6)	<.001	1.2 (0.8~2.0)	0.409
Alcohol drinker	1.2 (0.8~1.7)	0.329	0.9 (0.6~1.3)	0.484
eGFR (ml/min/l.73m2), per 10 unit increase	0.7 (0.6~0.8)	<.001	0.9 (0.8~1.0)	0.102
Interaction: Q2 of SUA * diabetes	1.7 (0.7~4.2)	0.239	10.1 (1.1~96.4)	0.044
Interaction: Q3 of SUA * diabetes	3.6 (1.9~6.8)	<.001	10.1 (1.2~86.0)	0.034
Interaction: Q4 of SUA * diabetes	2.6 (1.4~4.9)	0.002	4.6 (0.6~38.8)	0.158

Variables in multivariate logistic regression included all variables in the univariate logistic model. Other abbreviations are indicated in **Table 1**.

### Stratified Analyses of SUA and All-Cause Mortality in Diabetes and Non-Diabetes

**Figure 1:**
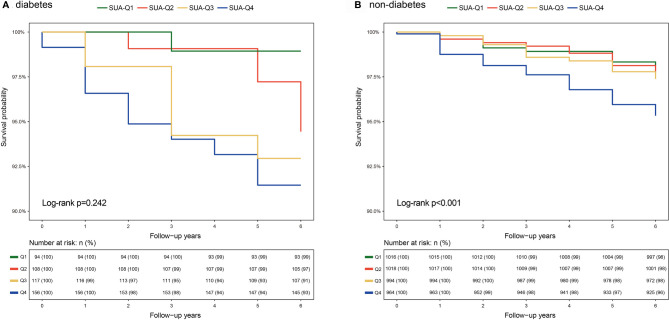
K-M curves of SUA quartiles for all-cause mortality among individuals with and without diabetes **(A)** Diabetic individuals, **(B)** Non-diabetic individuals. Abbreviations are indicated in **Table 1**).

K-M curve analysis was separately performed for participants with and without diabetes. The finding showed that participants with Q4 SUA exhibited higher all-cause mortality compared with participants with those lower SUA quartiles *(Log-rank P<0.001*, **Figure 1B**) in the non-diabetic group. However, the analysis did not show significant differences between SUA quartiles in the diabetic group *(Log-rank P=0.242*, **Figure 1A**). Findings from stratified analyses showed that Q1 and Q4 SUA levels in non-diabetic group were significantly positively associated with a risk of all-cause mortality compared with Q2 SUA levels (Q1 aHR=2.3, 95% CI 1.2~4.6, Q4 aHR=2.0, 95% CI 1.1~3.7) (**Table 3**). However, this effect of SUA quartiles was not observed among diabetic individuals.

**Table 3:** Hazard ratios of SUA levels for all-cause mortality among individuals with and without diabetes.

	rate per 1000 person-years	Univariable		age, gender-adjusted		Multivariable	
		HR (95% CI)	*P* value	HR (95% CI)	*P* value	HR (95% CI)	*P* value
Diabetes							
Q1 (Male: <4.7; Female: <3.7)	1.8 (1/561)	0.2 (0.1~2.0)	0.186	0.2 (0.1~1.9)	0.164	0.3 (0.1~2.3)	0.226
Q2 (Male: 4.7~5.5; Female: 3.7~4.4)	9.3 (6/642)	Ref (1.00)		Ref (1.00)		Ref (1.00)	
Q3 (Male: 5.6~6.9; Female: 4.5~5.4)	15.0 (10/666)	2.1 (0.7~6.1)	0.183	1.8 (0.6~5.3)	0.283	1.5 (0.5~4.4)	0.507
Q4 (Male:> 6.9; Female: >5.4)	12.2 (11/901)	1.5 (0.5~4.4)	0.429	1.3 (0.5~3.8)	0.616	0.9 (0.3~2.9)	0.900
Non-diabetes							
Q1 (Male: <4.7; Female: <3.7)	3.8 (23/6046)	1.3 (0.7~2.5)	0.391	2.0 (1.0~3.9)	0.039	2.3 (1.2~4.6)	0.015
Q2 (Male: 4.7~5.5; Female: 3.7~4.4)	3.1 (19/6055)	Ref (1.00)		Ref (1.00)		Ref (1.00)	
Q3 (Male: 5.6~6.9; Female: 4.5~5.4)	4.4 (26/5903)	1.5 (0.8~2.8)	0.202	1.3 (0.7~2.5)	0.377	1.4 (0.7~2.7)	0.288
Q4 (Male:> 6.9; Female: >5.4)	8.0 (45/5660)	2.7 (1.5~4.8)	<0.001	1.9 (1.0~3.4)	0.036	2.0 (1.1~3.7)	0.032

CI, confidence interval; HR, hazard ratio; Other abbreviations are indicated in **Table 1**. Multivariable HR was adjusted for age, gender, BMI, WHR, hypertension, dyslipidemia, smoking status and total protein intake (as 2nd quartile of SUA in non-diabetes individuals for reference).

Q1, male: <4.7 mg/dL or female: <3.7 mg/dL; Q2, male: 4.7~5.5 mg/dL or female: 3.7~4.4 mg/dL; Q3, male: 5.6~6.9 mg/dL or female: 4.5~5.4 mg/dL; Q4, male:> 6.9 mg/dL or female: >5.4 mg/dL.

### Multivariable Cox Regression Analysis of Diabetes and Non-Diabetes Participants

**Figure 2:**
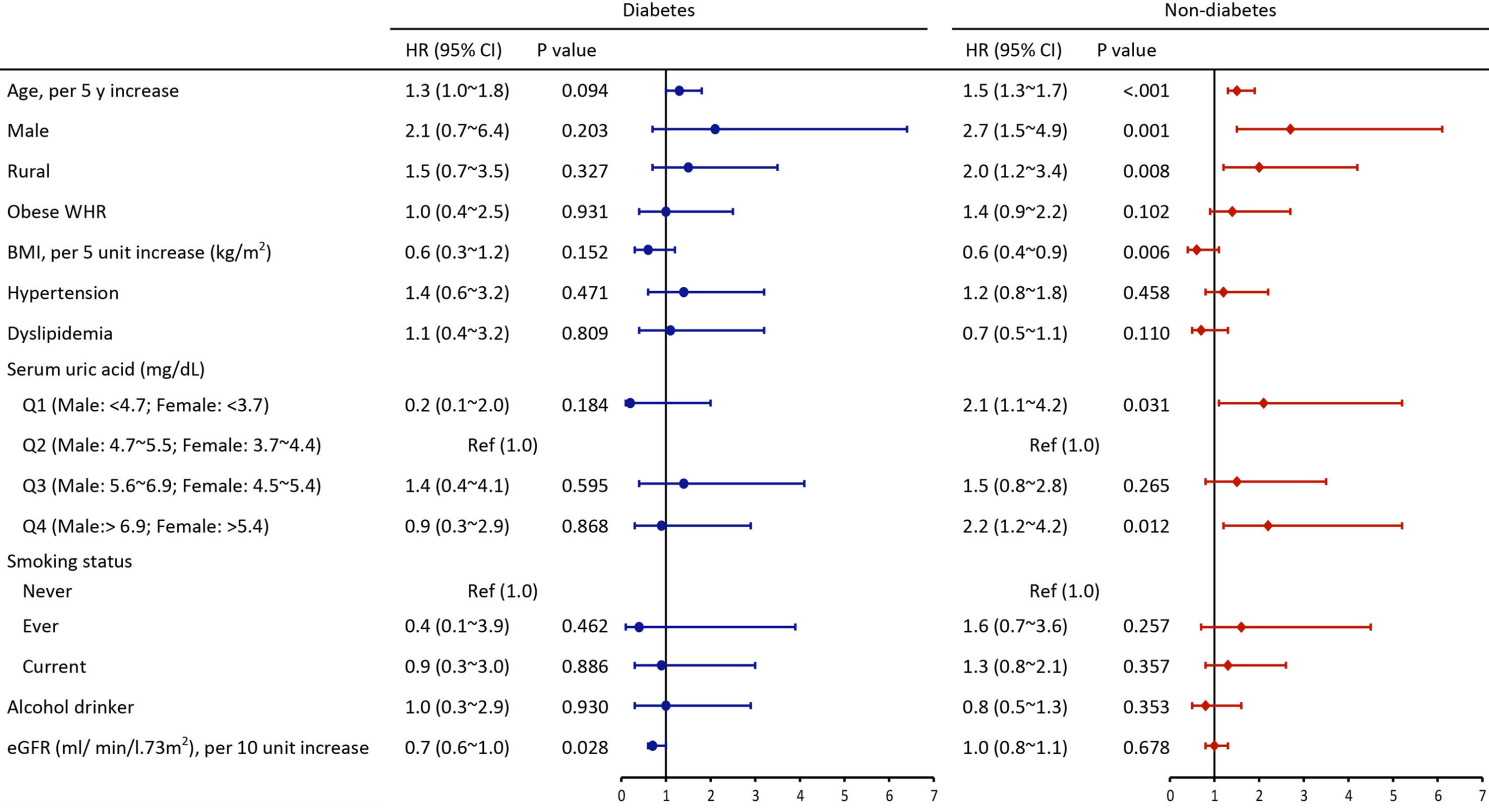
Multivariate adjusted hazard ratios of risk factors for all-cause mortality among individuals with and without diabetes (HR, hazard ratios; CI, confidence interval; Other abbreviations are indicated in **Table 1**).

All parameters were included in the multivariate Cox model to explore the most contributing factor. Analysis of the diabetes group showed that only low eGFR, rather than SUA level, was significantly associated with all-cause mortality (HR=0.7, 95%CI 0.6~1.0). Analysis of non-diabetic individuals showed that age (per 5-year increase: HR=1.5, 95%CI 1.3~1.7), male (HR=2.7, 95%CI 1.5~4.9), rural residence (HR=2.0, 95%CI 1.2~3.4), BMI (per 5 kg/m^2^ increase: HR=0.6, 95%CI 0.4~0.9), first and fourth quartile of SUA level (Q1 aHR= 2.1, 95%CI 1.1~4.2, Q4 aHR=2.2, 95%CI 1.2~4.2) were associated with risk of all-cause mortality (**Figure 2**).

### Sensitivity and Explanatory Analyses

Sensitivity analysis was conducted using MSM with inverse-probability weighting to test the robustness of the findings. A statistically significant association was observed between the fourth SUA quartile and all-cause death in non-diabetic individuals (aHR=2.7, 95%CI 1.5~4.8) (**Supplementary Table 5**). A total of 6 individuals who died in the baseline year were excluded from the second sensitivity analysis (**Supplementary Table 6**). In the third sensitivity analysis, if the event of all-cause death was recorded in the same year as baseline, follow-up duration was expressed as 0.5 years (**Supplementary Table 7**). Sensitivity analysis showed that the results remained robust. In addition, specified subgroup analyses showed significant associations between SUA quartiles and all-cause mortality were found in 45~59 years subgroup, non-hypertension subgroup, non-dyslipidemia subgroup, low FRS subgroup, and CKD subgroup (**Supplementary Table 8**).

## Discussion

The current study was a large population-based study with a 6 years follow-up period. The findings showed a U-shaped association between SUA and all-cause mortality among non-diabetic individuals. However, significant associations were not observed between SUA and all-cause mortality among diabetic individuals. Notably, the association between SUA and all-cause mortality was distinct between diabetes and non-diabetes participants. To the best of our knowledge, this is the first study that reports the association between SUA and all-cause mortality and the difference between diabetic and non-diabetic individuals in China.

The findings showed that all-cause mortality was 10.1/1000 person-years in diabetic group and 4.8/1000 person-years in non-diabetic group. This finding indicates that diabetic subjects have a 2.1-fold higher risk of death compared with non-diabetic participants. Estimates of all-cause mortality in the current study were similar to an estimation reported in a 7-year cohort study comprising 512,869 Chinese people aged 30-79 years (13.7 per 1000 person-years in diabetes and 6.5 per 1000 person-years in the non-diabetes [adjusted RR, 2.00 (95%CI, 1.93 to 2.08)] (32).

A cohort study conducted in China comprising 127 771 adults 65 years and older report higher CVD-related mortality for SUA level <4 mg/dL and ≥7 mg/dL, revealing a U-shaped association between SUA Levels with CVD and all-cause mortality (33). A previous meta-analysis reported that HUA was associated with increased risk of CHD morbidity (aRR 1.13; 95% CI 1.05~1.21) and mortality (aRR 1.27; 95% CI 1.16~1.39), with a dose-response in women (34). Of note, this association was mainly confounded by accompanied comorbidities. In addition, findings on association between SUA and mortality in the diabetic population are inconsistent. Lamacchia et al. reported a J-shaped relationship between SUA levels and all-cause mortality rate in patients with type 2 diabetes mellitus (19). On the contrary, Panero et al. reported that uric acid level was not an independent predictor for cardiovascular mortality in type 2 diabetes (35). This discrepancy can be attributed to differences in the study population, sample size, socioeconomic and cultural backgrounds. Most previous studies were limited by insufficient sample size, no appropriate comparisons, or narrow age range, thus limiting the ability to fully explore the independent effect of SUA levels on adverse renal outcomes. In the current study, similar linear associations were found among non-diabetic individuals but not in the diabetes group. This implies that the “U-shape” relationship between SUA levels and mortality may not be applicable to all individuals. This finding indicates that a higher mortality rate in diabetes was not attributable to SUA levels itself, thus it is consistent with previous findings that the mortality risk between diabetic and non-diabetic adults with high UA levels is not consistent (36).

Possible underlying mechanisms included in the pathogenesis of low-and-high SUA level-induced death. Low SUA is an indicator of poor nutritional status and vitamin C vitamin D deficiency, and malnourished status is an important factor in determining long-term survival (37–39). Extracellular UA is an antioxidant that can interact with hydrogen peroxide and hydroxyl radicals to effectively scavenge free radicals in the body, thus protecting vascular endothelial cells against oxidative attack. Also, the enzymatic reactions catalyzed by xanthine oxidase (hypoxanthine to xanthine and xanthine to uric acid) produce a lot of reactive species (ROS). Consequently, both low and high levels of SUA may cause the imbalance of redox state (40). However, high levels of soluble UA and urate crystals can induce the release of inflammatory chemokines, vascular cell muscle proliferation, fat synthesis in hepatocytes, oxidative stress, and decreased adiponectin synthesis in adipocytes, which are correlated with increased mortality (41, 42). Moreover, as the final product of adenosine metabolism, high adenosine plasma concentration is associated with uric acid concentrations in CAD patients, which may lead to a high risk of atrial fibrillation (43, 44).

Statistically significant association between SUA and all-cause mortality was observed only in non-diabetic individuals, implying that SUA levels play a significant role in non-diabetic subjects compared with diabetic adults. In contrast, all-cause death is mainly attributed to renal dysfunction among individuals with diabetes. Therefore, timely interventions in the early phases of prediabetes or even early stages of diabetes may be more effective compared with treatment at later stages, when immune system activation and target organ damage have occurred.

The role of SUA on all-cause mortality should be explored further but the results significantly depend on age, lipid metabolism, the follow-up duration and interplay between SUA and kidney function. Baseline analysis showed associations between SUA and other variables including obese WHR, hypertension, BMI, dyslipidemia and eGFR. These findings are consistent with findings from previous studies that HUA can reflect an underlying renal dysfunction, which is associated with a higher risk of death among diabetic individuals (45, 46). Moreover, changes in uric acid tubular reabsorption in presence of glycosuria can explain the inconsistent role of SUA (47, 48). Further mechanism studies should be conducted to explore the relationship between SUA on all-cause mortality.

A positive association between SUA and all-cause death among non-diabetic individuals was observed in the current study, indicating that SUA is an independent risk factor is strengthened. Significant differences in association of SUA with all-cause mortality observed in the non-diabetic subjects may indicate that SUA plays a larger role among individuals with low CVD risk. Subgroup analyses were conducted after stratification by age, BMI, WHR, hypertension, dyslipidemia, CKD and FRS. The findings showed significant associations between SUA and all-cause death for 45~59 years subgroup, non-hypertension subgroup, non-dyslipidemia subgroup, low FRS groups subgroup. These results further confirm our assumptions that SUA plays a larger role among individuals with low CVD risk. Therefore, target comprehensive treatment should be used to manage risk factors in respective groups. Studies should explore the timely treatment of patients with the abnormal value of SUA with routine urate-lowering therapy (ULT) owing to the disparity of relationship between SUA and death under different disease backgrounds. Studies that are exploring this relationship and effects of therapies at different times are underway.

Some limitations deserve mention. Firstly, analyses were based on baseline SUA levels and its stability during the follow-up period of 5.9 years cannot be definitively determined. Secondly, the cause of death was not reported in the current study. Previous studies with long follow-up periods report that HUA can be an early manifestation of the carcinogenic process and CVD death, thus the association between uric acid, cancer and CVD risk should be explored in further studies (49, 50). Thirdly, the smaller sample size of diabetic individuals can lead to bias in results obtained from analyses across SUA categories. Moreover, differences in all-cause death between the two groups were satisfactory, as only a few events were reported and the wide confidence intervals of estimates were used. Lastly, concomitant medications including ULT, anti-hypertensive agents and diuretics may affect the risk of cardiovascular events and all-cause mortality, however, treatment information was not collected for all participants included in the CHNS study (14, 51, 52).

## Conclusion

Our study proved that the prognostic role of SUA for predicting all-cause death might be regulated by diabetes. Both low and high SUA levels were associated with increased mortality, supporting a U-shaped association only in non-diabetic individuals. Whereas, renal dysfunction rather than SUA was an independent risk factor for all-cause mortality. Further studies should be conducted to determine the SUA levels at which intervention should be conducted and explore target follow-up strategies to prevent progression leading to poor prognosis.

## Data Availability Statement

Publicly available datasets were analyzed in this study. This data can be found here: https://www.cpc.unc.edu/projects/china/data/datasets/data-downloads-registration.

## Ethics Statement

CHNS was approved by the Institutional Review Board at the University of North Carolina at Chapel Hill and local IRB (institutional review board or ethics committee). The patients/participants provided their written informed consent to participate in this study.

## Author Contributions

BZ and XD contributed to the conception or design of the work. BZ, JZ, and YL contributed to the acquisition, analysis, or interpretation of data for the work. BZ and YL drafted the manuscript. YL and YF critically revised the manuscript. BZ, JZ, NS, YS, YF, YL, and XD contribute to analysis, or interpretation of the work. All authors contributed to the article and approved the submitted version.

## Funding

Apart from the original grants to the CHNS, this study was sponsored by the Natural Science Foundation of Shanghai (21ZR1412400), National Natural Science Foundation of China (8210120481 and 81870476), Shanghai Key Laboratory of Kidney and Blood Purification (14DZ2260200), Shanghai Science and Technology Commission (18411960800), Innovation Program of Shanghai Municipal Education Commission (2017-01-07-00-07-E00009), and Shanghai Municipal Key Clinical Specialty (shslczdzk02501).

## Conflict of Interest

The authors declare that the research was conducted in the absence of any commercial or financial relationships that could be construed as a potential conflict of interest.

## Publisher’s Note

All claims expressed in this article are solely those of the authors and do not necessarily represent those of their affiliated organizations, or those of the publisher, the editors and the reviewers. Any product that may be evaluated in this article, or claim that may be made by its manufacturer, is not guaranteed or endorsed by the publisher.

## Abbreviations

AKI, acute kidney injury; BMI, body mass index; BP, blood pressure; CCDC, Chinese Center for Disease Control and Prevention; CHNS, China Health and Nutrition Survey; CI, confidence interval; CKD, chronic kidney disease; CVD, cardiovascular disease; DKD, diabetic kidney disease; eGFR, estimated glomerular filtration rate; HUA, hyperuricemia; HR, hazard ratios; LDL, low-density lipoprotein cholesterol; IQR, interquartile range; MI, myocardial infarction; SD, standard deviation; SUA, serum uric acid; MSM, marginal structure model; WHR, waist to hip circumference ratio; OR, odds ratio; RR, risk ratio; SD, standard drink; ULT, urate-lowering therapy.
