# Supplementary material for: Distinct Prognostic Role of Serum Uric Acid Levels for Predicting All-Cause Mortality Among Chinese Adults Aged 45~75 Years With and Without Diabetes
Source: Front Endocrinol (Lausanne). 2021 Nov 16;12:782230. doi: 10.3389/fendo.2021.782230 (PMC8636854; doi:10.3389/fendo.2021.782230)
Supplement: Supplementary file 1 [file DataSheet_1.docx]

**Supplementary data**

**Supplementary Figure 1.** Flowchart showing the study population selection process and showing the number of subjects excluded owing to different reasons

**Supplementary Table 1.** Characteristics of participants included in the analysis and those excluded from the final analysis

**Supplementary Table 2.** Baseline characteristics of individuals in CHNS cohorts with and without diabetes

**Supplementary Table 3.** Risk ratio of SUA levels for all-cause mortality among individuals with and without diabetes

**Supplementary Table 4.** Hazard ratios of risk factors for all-cause mortality among all individuals

**Supplementary Table 5.** Sensitivity analysis of hazard ratios of SUA levels for all-cause mortality among individuals with and without diabetes, using MSM model

**Supplementary Table 6.** Sensitivity analysis of hazard ratios of risk factors for all-cause mortality among individuals with and without diabetes, deleting individuals who died at 2009 (n=4463)

**Supplementary Table 7.** Sensitivity analysis of hazard ratios of risk factors for all-cause mortality among individuals with and without diabetes, counting follow-up duration of individuals who died in 2009 as 0.5 years

**Supplementary Table 8.** Association between SUA levels and all-cause mortality in 45~75 years participants, stratified by age, gender, WHR, hypertension, dyslipidemia, CKD and FRS

**Supplementary Table 9.** Detailed information on the diabetes

**Supplementary Table 10.** All-cause mortality between treatment groups among individuals with diabetes history


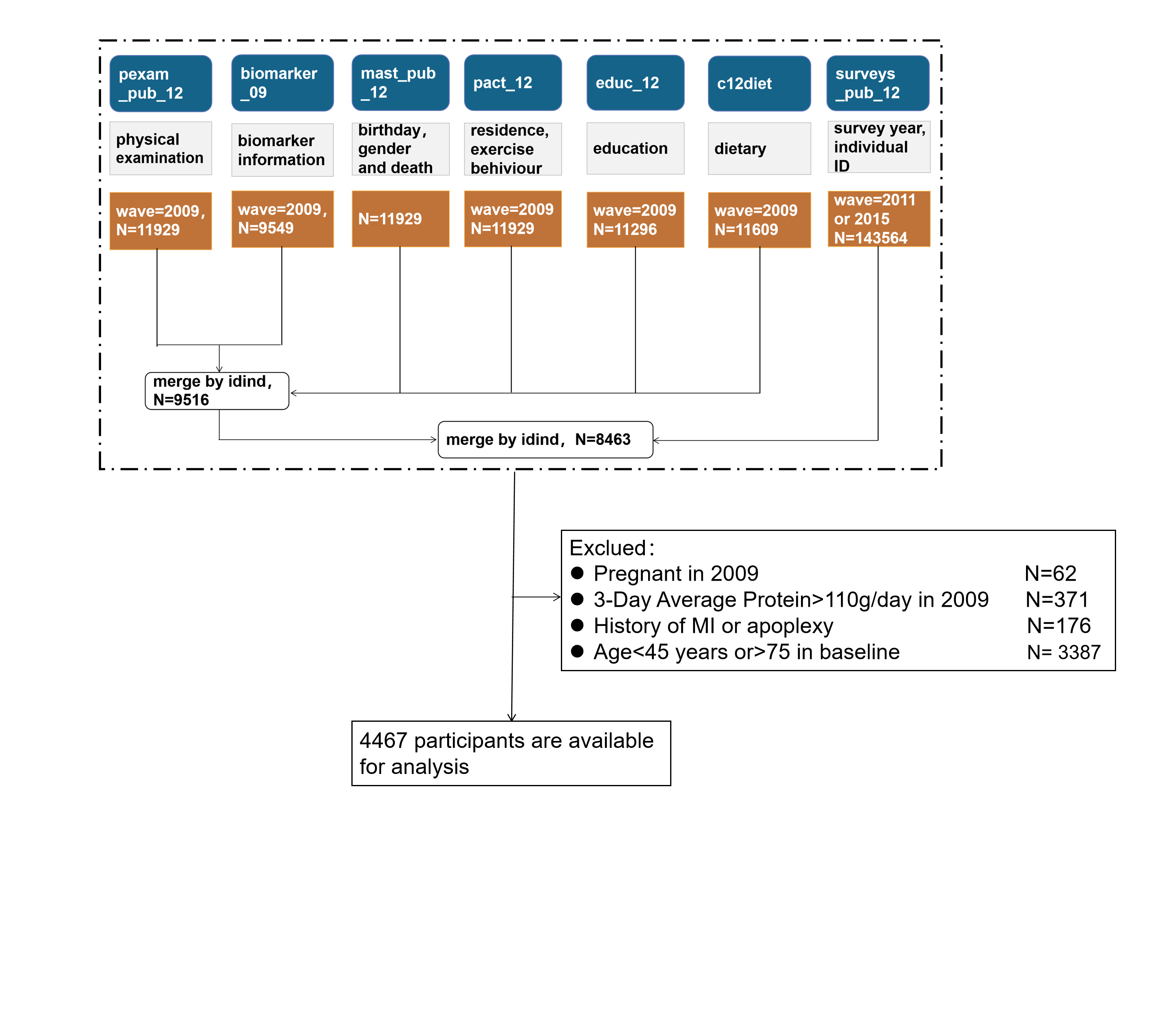


Supplementary Figure 1. Flowchart showing the study population selection process and showing the number of subjects excluded owing to different reasons

Supplementary Table1. Characteristics of participants included in the analysis and those excluded from the final analysis

|  | Analytic Sample | Missing sample | *P-*value*** |
| --- | --- | --- | --- |
| Participants (n) | 4467 | 378 |  |
| Age (years) | 57.7 (8.3) | 57.6 (8.2) | 0.845 |
| Male (%) | 2024 (45.3) | 171 (45.2) | 0.976 |
| Education (years) |  |  | 0.004 |
| 0 | 694 (15.6) | 69 (18.3) |  |
| ≤6 | 1539 (34.5) | 96 (25.4) |  |
| 7–9 | 1313 (29.4) | 106 (28.0) |  |
| 10–12 | 517 (11.6) | 62 (16.4) |  |
| >12 | 397 (8.9) | 45 (11.9) |  |
| Rural (%) | 3051 (68.3) | 163 (43.1) | <0.001 |
| *Anthropometry parameters* |  |  |  |
| Waist (cm) | 84.1 (10.0) | 84.1 (10.0) | 0.957 |
| Hip (cm) | 95.0 (7.8) | 96.1 (7.7) | 0.009 |
| Obese WHR | 2418 (55.5) | 185 (50.4) | 0.089 |
| BMI (kg/m2) |  |  | 0.006 |
| Lean (<18.5) | 219 (4.9) | 14 (3.7) |  |
| Normal (18.5–23.9) | 2300 (51.5) | 185 (48.9) |  |
| Overweight (24–27.9) | 1485 (33.2) | 125 (33.1) |  |
| Obesity (≥28) | 463 (10.4) | 54 (14.3) |  |
| Systolic BP (mm Hg) | 128.1 (18.7) | 128.9 (18.1) | 0.452 |
| Diastolic BP (mm Hg) | 82 (11.2) | 81.9 (10.4) | 0.763 |
| Hypertension | 1372 (34.6) | 120 (35.6) | 0.695 |
| Diabetes | 475 (10.6) | 52 (13.8) | 0.061 |
| Dyslipidemia | 2966 (66.4) | 255 (67.5) | 0.674 |
| SUA (mg/dL) | 5.2 (1.7) | 5.3 (1.8) | 0.313 |
| eGFR (ml/ min/l.73m^2^) | 74.6 (13.8) | 74 (14.3) | 0.398 |
| Framingham score (%) | 9.4 (5.3~11.9) | 9.4 (5.3~18.4) | 0.308 |
| *Health-related behavior* |  |  |  |
| Smoking status |  |  | 0.944 |
| Never | 3031 (67.9) | 254 (67.2) |  |
| Ever | 154 (3.5) | 14 (3.7) |  |
| Current | 1279 (28.7) | 110 (29.1) |  |
| Alcohol drinker | 1444 (32.3) | 135 (35.7) | 0.178 |
| Total protein intake (g/day) | 62.8 (18.8) | 64 (19.4) | 0.253 |

Abbreviation: BMI, body mass index; BP, blood pressure; eGFR, estimated glomerular filtration rate; MI, myocardial infarction; SUA, serum uric acid; WHR, waist to hip circumference ratio.

Data are presented as No. (%), mean± SD or median (IQR);

*P values were calculated by using student t-test or Wilcoxon test for continuous variables and χ^2^ test or Fisher exact test for categorical variables.

7 participants were not available for education level; 121 participants were not available for WHR; 537 participants were not available for hypertension; 3 participants were not available for smoking status; 1 participant was not available for drinking behavior.

Supplementary Table 2. Baseline characteristics of individuals in CHNS cohorts with and without diabetes

|  | Diabetes | Non-diabetes | *P-*value* |
| --- | --- | --- | --- |
| Participants (n) | 475 | 3992 |  |
| All cause mortality (%) | 28 (5.9) | 113 (2.8) | <0.001 |
| Age (years) | 59.9 (8.7) | 57.4 (8.2) | <0.001 |
| Male (%) | 218 (45.9) | 1806 (45.2) | 0.787 |
| Education (years) |  |  | 0.333 |
| 0 | 67 (14.1) | 627 (15.7) |  |
| ≤6 | 167 (35.2) | 1372 (34.4) |  |
| 7–9 | 139 (29.3) | 1174 (29.5) |  |
| 10–12 | 49 (10.3) | 468 (11.7) |  |
| >12 | 53 (11.2) | 344 (8.6) |  |
| Rural (%) | 290 (61.1) | 2761 (69.2) | <0.001 |
| *Anthropometry parameters* |  |  |  |
| Waist (cm) | 90.5 (9.8) | 83.4 (9.8) | <0.001 |
| Hip (cm) | 99 (7.6) | 94.6 (7.7) | <0.001 |
| Obese WHR | 350 (76.1) | 2068 (53.1) | <0.001 |
| BMI (kg/m^2^) |  |  | <0.001 |
| Lean (<18.5) | 8 (1.7) | 211 (5.3) |  |
| Normal (18.5–23.9) | 154 (32.4) | 2146 (53.8) |  |
| Overweight (24–27.9) | 199 (41.9) | 1286 (32.2) |  |
| Obesity (≥28) | 114 (24.0) | 349 (8.7) |  |
| Systolic BP (mm Hg) | 134.4 (17.1) | 127.4 (18.7) | <0.001 |
| Diastolic BP (mm Hg) | 84 (10.9) | 81.8 (11.2) | <0.001 |
| Hypertension | 222 (50.0) | 1150 (32.6) | <0.001 |
| Diabetes |  |  |  |
| Serum uric acid (mg/dL) |  |  | <0.001 |
| Q1 (Male: <4.7; Female: <3.7) | 94 (19.8) | 1016 (25.5) |  |
| Q2 (Male: 4.7~5.5; Female: 3.7~4.4) | 108 (22.7) | 1018 (25.5) |  |
| Q3 (Male: 5.6~6.9; Female: 4.5~5.4) | 117 (24.6) | 994 (24.9) | <0.001 |
| Q4 (Male:> 6.9; Female: >5.4) | 156 (32.8) | 964 (24.1) |  |
| Dyslipidemia | 381 (80.2) | 2585 (64.8) | <0.001 |
| eGFR (ml/ min/l.73m^2^) | 72.2 (16.3) | 74.9 (13.4) | <0.001 |
| Framingham score (%) | 18.4 (11.7~29.4) | 8.6 (5.3~15.6) | <0.001 |
| *Health-related behavior* |  |  |  |
| Smoking status |  |  | 0.016 |
| Never | 319 (67.3) | 2712 (68.0) |  |
| Ever | 27 (5.7) | 127 (3.2) |  |
| Current | 128 (27.0) | 1151 (28.9) |  |
| Alcohol drinker | 154 (32.4) | 1290 (32.3) | 0.996 |
| Total protein intake (g/day) | 62.8 (18.9) | 62.8 (18.8) | 0.969 |
| History of diabetes | 166 (35.0) | - | - |
| HbA1c ≥6.5% | 410 (86.3) | - | - |

Abbreviations are indicated in Supplementary Table 1. Data are presented as No. (%), mean±SD or median (IQR); *P values were calculated by using student t-test or Wilcoxon test for continuous variables and χ^2^ test or Fisher exact test for categorical variables.

7 participants were not available for education level; 121 participants were not available for WHR; 537 participants were not available for hypertension; 3 participants were not available for smoking status; 1 participant was not available for drinking behavior

Supplementary Table 3. Risk ratio of SUA levels for all-cause mortality among individuals with and without diabetes

|  | Prevalence rate, % | Univariable | |  | age, gender-adjusted | |  | Multivariable | |
| --- | --- | --- | --- | --- | --- | --- | --- | --- | --- |
|  |  | RR (95% CI) | *P*-value |  | RR (95% CI) | *P-*value |  | RR (95% CI) | *P*-value |
| All participants |  |  |  |  |  |  |  |  |  |
| Q1, non-diabetes | 2.3 (23/1016) | 1.3 (0.7~2.6) | 0.388 |  | 1.9 (1.0~3.8) | 0.055 |  | 2.3 (1.2~4.6) | 0.017 |
| Q2, non-diabetes | 1.9 (19/1018) | Ref (1.00) | |  | Ref (1.00) | |  | Ref (1.00) | |
| Q3, non-diabetes | 2.6 (26/994) | 1.5 (0.8~2.9) | 0.200 |  | 1.4 (0.7~2.6) | 0.342 |  | 1.4 (0.7~2.7) | 0.321 |
| Q4, non-diabetes | 4.7 (45/964) | 2.7 (1.5~4.9) | <.001 |  | 2.0 (1.1~3.7) | 0.021 |  | 1.9 (1.0~3.7) | 0.041 |
| Q1, diabetes | 1.1 (1/94) | 0.6 (0.1~4.9) | 0.668 |  | 0.6 (0.1~4.4) | 0.594 |  | 0.7 (0.1~5.3) | 0.712 |
| Q2, diabetes | 5.6 (6/108) | 2.8 (1.0~7.9) | 0.047 |  | 2.7 (0.9~7.6) | 0.064 |  | 3.1 (1.1~9.1) | 0.034 |
| Q3, diabetes | 8.5 (10/117) | 5.9 (2.6~13.4) | <.001 |  | 4.8 (2.1~11.1) | <.001 |  | 4.7 (2.0~11.1) | <.001 |
| Q4, diabetes | 7.1 (11/156) | 4.4 (2.0~9.6) | <.001 |  | 3.1 (1.4~7.0) | 0.006 |  | 3.1 (1.3~7.2) | 0.010 |
| Diabetes |  |  |  |  |  |  |  |  |  |
| Q1 | 1.1 (1/94) | 0.2 (0.0~2.0) | 0.179 |  | 0.2 (0~1.8) | 0.155 |  | 0.3 (0.0~2.3) | 0.219 |
| Q2 | 5.6 (6/108) | Ref (1.00) | |  | Ref (1.00) | |  | Ref (1.00) | |
| Q3 | 8.5 (10/117) | 2.1 (0.7~6.3) | 0.194 |  | 1.8 (0.6~5.7) | 0.299 |  | 1.4 (0.4~4.7) | 0.557 |
| Q4 | 7.1 (11/156) | 1.5 (0.5~4.6) | 0.439 |  | 1.3 (0.4~4) | 0.639 |  | 0.9 (0.3~2.9) | 0.835 |
| Non-diabetes |  |  |  |  |  |  |  |  |  |
| Q1 | 2.3 (23/1016) | 1.3 (0.7~2.6) | 0.388 |  | 2.0 (1.0~4.0) | 0.040 |  | 2.4 (1.2~4.8) | 0.016 |
| Q2 | 1.9 (19/1018) | Ref (1.00) | |  | Ref (1.00) | |  | Ref (1.00) | |
| Q3 | 2.6 (26/994) | 1.5 (0.8~2.9) | 0.200 |  | 1.3 (0.7~2.6) | 0.375 |  | 1.4 (0.7~2.8) | 0.292 |
| Q4 | 4.7 (45/964) | 2.7 (1.5~4.9) | <.001 |  | 1.9 (1.1~3.5) | 0.031 |  | 2.1 (1.1~3.9) | 0.029 |

Abbreviations: CI, confidence interval; RR, risk ratio; Other abbreviations are indicated in Supplementary Table 1.

Multivariable RR was adjusted for age, gender, BMI, WHR, hypertension, dyslipidemia, smoking status, and total protein intake.

Logistic regression analyses and MSM model of SUA with the incidence of all-cause death by SUA group (as 2nd quartile of SUA in non-diabetes individuals for reference).

Supplementary Table 4. Hazard ratios of risk factors for all-cause mortality among all individuals

|  | Univariable | |  | Mulvariable | |
| --- | --- | --- | --- | --- | --- |
|  | HR (95% CI) | *P* value |  | HR (95% CI) | *P* value |
| Age, per 5 y increase | 1.6 (1.4~1.8) | <.001 |  | 1.4 (1.3~1.6) | <.001 |
| Male | 2.3 (1.6~3.2) | <.001 |  | 2.5 (1.5~4.1) | <.001 |
| Rural | 1.6 (1.1~2.4) | 0.017 |  | 1.9 (1.2~2.9) | 0.004 |
| Obese WHR | 1.1 (0.8~1.6) | 0.453 |  | 1.3 (0.9~1.9) | 0.196 |
| BMI, per 5 unit increase (kg/m^2^) | 0.6 (0.5~0.8) | <.001 |  | 0.6 (0.5~0.8) | 0.002 |
| Hypertension | 1.7 (1.2~2.4) | 0.003 |  | 1.3 (0.9~1.8) | 0.230 |
| Diabetes | 2.2 (1.4~3.3) | <.001 |  | 0.4 (0.1~2.7) | 0.324 |
| Dyslipidemia | 0.8 (0.6~1.2) | 0.286 |  | 0.8 (0.5~1.2) | 0.242 |
| Serum uric acid (mg/dL) |  |  |  |  |  |
| Q1 (Male: <4.7; Female: <3.7) | 1.1 (0.6~2) | 0.811 |  | 2.1 (1.1~4.1) | 0.031 |
| Q2 (Male: 4.7~5.5; Female: 3.7~4.4) | Ref (1.00) | |  | Ref (1.00) | |
| Q3 (Male: 5.6~6.9; Female: 4.5~5.4) | 1.6 (0.9~2.8) | 0.077 |  | 1.4 (0.7~2.7) | 0.290 |
| Q4 (Male:> 6.9; Female: >5.4) | 2.5 (1.5~4.1) | <.001 |  | 2.1 (1.1~4.0) | 0.018 |
| Smoking status |  |  |  |  |  |
| Never | Ref (1.00) | |  | Ref (1.00) | |
| Ever | 3.3 (1.8~6.1) | <.001 |  | 1.2 (0.6~2.6) | 0.625 |
| Current | 1.8 (1.3~2.6) | <.001 |  | 1.2 (0.8~2.0) | 0.409 |
| Alcohol drinker | 1.2 (0.8~1.7) | 0.329 |  | 0.9 (0.6~1.3) | 0.484 |
| eGFR (ml/ min/l.73m2), per 10 unit increase | 0.7 (0.6~0.8) | <.001 |  | 0.9 (0.8~1.0) | 0.102 |
| Interaction: Q2 of SUA * diabetes | 1.7 (0.7~4.2) | 0.239 |  | 10.1 (1.1~96.4) | 0.044 |
| Interaction: Q3 of SUA * diabetes | 3.6 (1.9~6.8) | <.001 |  | 10.1 (1.2~86.0) | 0.034 |
| Interaction: Q4 of SUA * diabetes | 2.6 (1.4~4.9) | 0.002 |  | 4.6 (0.6~38.8) | 0.158 |

Abbreviations are indicated in Supplementary Table 1. Variables in multivariate logistic regression included all variables in the univariate model.

Supplementary Table 5. Sensitivity analysis of hazard ratios of SUA levels for all-cause mortality among individuals with and without diabetes using MSM model

|  | MSM model | |
| --- | --- | --- |
|  | HR (95% CI) | *P* value |
| Diabetes |  |  |
| Q1 (Male: <4.7; Female: <3.7) | 0.2 (0.1~2.0) | 0.186 |
| Q2 (Male: 4.7~5.5; Female: 3.7~4.4) | Ref (1.00) | |
| Q3 (Male: 5.6~6.9; Female: 4.5~5.4) | 2.1 (0.7~6.1) | 0.183 |
| Q4 (Male:> 6.9; Female: >5.4) | 1.5 (0.5~4.4) | 0.429 |
| Non-diabetes |  |  |
| Q1 (Male: <4.7; Female: <3.7) | 1.3 (0.7~2.5) | 0.391 |
| Q2 (Male: 4.7~5.5; Female: 3.7~4.4) | Ref (1.00) | |
| Q3 (Male: 5.6~6.9; Female: 4.5~5.4) | 1.5 (0.8~2.8) | 0.202 |
| Q4 (Male:> 6.9; Female: >5.4) | 2.7 (1.5~4.8) | <0.001 |

Abbreviation: HR, Hazards ratios; CI, confidence interval; MSM, marginal structure model; other abbreviations are indicated in Supplementary Table 1.

Supplementary Table 6. Sensitivity analysis of hazard ratios of risk factors for all-cause mortality among individuals with and without diabetes, excluding individuals who died in 2009 (n=4463)

|  | Diabetes | | | | |  | Non-diabetes | | | | |
| --- | --- | --- | --- | --- | --- | --- | --- | --- | --- | --- | --- |
|  | Univariable | |  | Mulvariable | |  | Univariable | |  | Mulvariable | |
|  | HR (95% CI) | *P v*alue |  | HR (95% CI) | *P* value |  | HR (95% CI) | *P* value |  | HR (95% CI) | *P* value |
| Age, per 5 y increase | 1.5 (1.2~1.9) | 0.001 |  | 1.4 (1.0~1.9) | 0.054 |  | 1.6 (1.4~1.8) | <.001 |  | 1.5 (1.3~1.7) | <.001 |
| Male | 1.3 (0.6~2.7) | 0.514 |  | 2.2 (0.7~7.0) | 0.180 |  | 2.8 (1.9~4.3) | <.001 |  | 2.9 (1.6~5.3) | <.001 |
| Rural | 1.1 (0.5~2.3) | 0.864 |  | 1.5 (0.6~3.4) | 0.371 |  | 1.9 (1.2~3.1) | 0.009 |  | 1.9 (1.1~3.3) | 0.014 |
| Obese WHR | 0.9 (0.4~2.2) | 0.901 |  | 0.9 (0.3~2.4) | 0.810 |  | 1.1 (0.8~1.6) | 0.553 |  | 1.6 (1.0~2.4) | 0.053 |
| BMI, per 5 unit increase (kg/m^2^) | 0.6 (0.4~1.1) | 0.126 |  | 0.7 (0.4~1.3) | 0.238 |  | 0.6 (0.4~0.8) | 0.0008 |  | 0.7 (0.5~1.0) | 0.026 |
| Hypertension | 1.9 (0.8~4.2) | 0.121 |  | 1.3 (0.5~3.1) | 0.547 |  | 1.6 (1.1~2.4) | 0.023 |  | 1.2 (0.8~1.9) | 0.355 |
| Dyslipidemia | 0.9 (0.4~2.2) | 0.771 |  | 1.1 (0.4~3.1) | 0.860 |  | 0.7 (0.5~1.1) | 0.122 |  | 0.7 (0.5~1.1) | 0.106 |
| Serum uric acid (mg/dL) |  |  |  |  |  |  |  |  |  |  |  |
| Q1 (Male: <4.7; Female: <3.7) | 0.2 (0.1~2.0) | 0.186 |  | 0.2 (0.1~2.0) | 0.177 |  | 1.4 (0.7~2.6) | 0.379 |  | 2.1 (1.1~4.3) | 0.036 |
| Q2 (Male: 4.7~5.5; Female: 3.7~4.4) | Ref (1.00) | |  | Ref (1.00) | |  | Ref (1.00) | |  | Ref (1.00) | |
| Q3 (Male: 5.6~6.9; Female: 4.5~5.4) | 1.9 (0.6~5.6) | 0.261 |  | 1.3 (0.4~4.0) | 0.697 |  | 1.6 (0.8~3.1) | 0.148 |  | 1.6 (0.8~3.1) | 0.187 |
| Q4 (Male:> 6.9; Female: >5.4) | 1.5 (0.5~4.4) | 0.428 |  | 0.9 (0.3~3.0) | 0.910 |  | 2.8 (1.5~5.1) | <.001 |  | 2.4 (1.3~4.6) | 0.008 |
| Smoking status |  |  |  |  |  |  |  |  |  |  |  |
| Never | Ref (1.00) | |  | Ref (1.00) | |  | Ref (1.00) | |  | Ref (1.00) | |
| Ever | 0.6 (0.1~4.9) | 0.674 |  | 0.4 (0.1~3.5) | 0.415 |  | 4.2 (2.1~8.2) | <.001 |  | 1.4 (0.6~3.2) | 0.481 |
| Current | 1.3 (0.6~3.0) | 0.471 |  | 0.9 (0.3~3.0) | 0.905 |  | 2.1 (1.4~3.1) | <.001 |  | 1.3 (0.8~2.1) | 0.364 |
| Alcohol drinker | 0.8 (0.4~1.9) | 0.648 |  | 0.9 (0.3~2.7) | 0.850 |  | 1.4 (0.9~2.0) | 0.117 |  | 0.8 (0.5~1.3) | 0.320 |
| eGFR (ml/ min/l.73m2), per 10 unit increase | 0.7 (0.5~0.8) | <.001 |  | 0.8 (0.6~1.0) | 0.085 |  | 0.8 (0.7~0.9) | <.001 |  | 1.0 (0.8~1.2) | 0.690 |

Abbreviations are indicated in Supplementary Table 1. Variables in multivariate logistic regression included all variables in the univariate model.

Supplementary Table 7. Sensitivity analysis of hazard ratios of risk factors for all-cause mortality among individuals with and without diabetes, including follow-up duration of individuals who died in 2009 as 0.5 years

|  | Diabetes | | | | |  | Non-diabetes | | | | |
| --- | --- | --- | --- | --- | --- | --- | --- | --- | --- | --- | --- |
|  | Univariable | |  | Mulvariable | |  | Univariable | |  | Mulvariable | |
|  | HR (95% CI) | *P* value |  | HR (95% CI) | *P* value |  | HR (95% CI) | *P* value |  | HR (95% CI) | *P* value |
| Age, per 5 y increase | 1.5 (1.2~1.9) | 0.001 |  | 1.3 (1.0~1.8) | 0.094 |  | 1.6 (1.4~1.8) | <.001 |  | 1.5 (1.3~1.7) | <.001 |
| Male | 1.2 (0.6~2.5) | 0.640 |  | 2.1 (0.7~6.7) | 0.203 |  | 2.7 (1.8~4.0) | <.001 |  | 2.6 (1.4~4.8) | 0.002 |
| Rural | 1.1 (0.5~2.5) | 0.749 |  | 1.5 (0.7~3.5) | 0.462 |  | 2.0 (1.2~3.2) | 0.006 |  | 2.0 (1.2~3.4) | 0.010 |
| Obese WHR | 1.0 (0.4~2.3) | 0.986 |  | 0.9 (0.3~2.5) | 0.931 |  | 1.1 (0.7~1.5) | 0.778 |  | 1.5 (0.9~2.3) | 0.088 |
| BMI, per 5 unit increase (kg/m^2^) | 0.6 (0.3~1.1) | 0.075 |  | 0.6 (0.3~1.2) | 0.152 |  | 0.6 (0.4~0.8) | <.001 |  | 0.6 (0.4~0.9) | 0.014 |
| Hypertension | 2.0 (0.9~4.5) | 0.088 |  | 1.4 (0.6~3.2) | 0.471 |  | 1.5 (1.0~2.2) | 0.042 |  | 1.2 (0.8~1.8) | 0.444 |
| Dyslipidemia | 0.9 (0.4~2.3) | 0.848 |  | 1.1 (0.4~3.2) | 0.327 |  | 0.8 (0.5~1.1) | 0.130 |  | 0.7 (0.5~1.1) | 0.137 |
| Serum uric acid (mg/dL) |  |  |  |  |  |  |  |  |  |  |  |
| Q1 (Male: <4.7; Female: <3.7) | 0.2 (0.1~2) | 0.186 |  | 0.2 (0.1~2.0) | 0.184 |  | 1.3 (0.7~2.5) | 0.391 |  | 2.1 (1.1~4.1) | 0.036 |
| Q2 (Male: 4.7~5.5; Female: 3.7~4.4) | Ref (1.00) | |  | Ref (1.00) | |  | Ref (1.00) | |  | Ref (1.00) | |
| Q3 (Male: 5.6~6.9; Female: 4.5~5.4) | 2.1 (0.7~6.1) | 0.183 |  | 1.3 (0.4~4.1) | 0.595 |  | 1.5 (0.8~2.8) | 0.202 |  | 1.5 (0.8~2.8) | 0.242 |
| Q4 (Male:> 6.9; Female: >5.4) | 1.5 (0.5~4.4) | 0.429 |  | 0.9 (0.3~2.8) | 0.868 |  | 2.7 (1.5~4.8) | <.001 |  | 2.4 (1.2~4.4) | 0.008 |
| Smoking status |  |  |  |  |  |  |  |  |  |  |  |
| Never | Ref (1.00) | |  | Ref (1.00) | |  | Ref (1.00) | |  | Ref (1.00) | |
| Ever | 0.6 (0.1~4.6) | 0.635 |  | 0.4 (0.1~3.9) | 0.886 |  | 4.4 (2.3~8.4) | <.001 |  | 1.6 (0.7~3.5) | 0.289 |
| Current | 1.3 (0.6~2.8) | 0.557 |  | 0.9 (0.3~2.9) | 0.930 |  | 2.0 (1.3~2.9) | <.001 |  | 1.3 (0.7~2.1) | 0.387 |
| Alcohol drinker | 0.8 (0.3~1.8) | 0.564 |  | 0.9 (0.3~2.9) | 0.993 |  | 1.3 (0.9~1.9) | 0.175 |  | 0.8 (0.5~1.3) | 0.347 |
| eGFR (ml/ min/l.73m2), per 10 unit increase | 0.6 (0.5~0.8) | <.001 |  | 0.7 (0.6~1.0) | 0.028 |  | 0.8 (0.7~0.9) | <.001 |  | 1.0 (0.8~1.1) | 0.647 |

Abbreviations are indicated in Supplementary Table 1. Variables in multivariate logistic regression included all variables in the univariate model.

Supplementary Table 8. Association between SUA levels and all-cause mortality in 45~75 years participants, stratified by age, BMI, WHR, hypertension, dyslipidemia, CKD and FRS

|  | No. of Death/1000 Person-Years | | | |  | Age and gender-adjusted HR (95% CI) | | | |
| --- | --- | --- | --- | --- | --- | --- | --- | --- | --- |
|  | Q1 (Male: <4.7 mg/dL; Female: <3.7mg/dL) | Q2 (Male: 4.7~5.5mg/d; Female: 3.7~4.4mg/d) | Q3 (Male: 5.6~6.9mg/d; Female: 4.5~5.4mg/d) | Q4 (Male:  > 6.9 mg/dL; Female:  >5.4 mg/dL) |  | Q1 (Male:  <4.7 mg/dL; Female: <3.7mg/dL) | Q2 (Male: 4.7~5.5mg/dL; Female: 3.7~4.4mg/d) | Q3 (Male: 5.6~6.9mg/dL; Female:  4.5~5.4mg/L) | Q4 (Male:  > 6.9 mg/dL; Female:  >5.4 mg/dL) |
| Age, years |  |  |  |  |  |  |  |  |  |
| 45~59 | 1.1 (5/4427) | 1.2 (5/4085) | 2.3 (9/3841) | 5.1 (19/3727) |  | 1.1 (0.3~3.9) | 1.00 (ref) | 1.8 (0.6~5.3) | 3.5 (1.3~9.7)^*^ |
| 60~75 | 8.7 (19/2180) | 7.7 (20/2612) | 9.9 (27/2728) | 13.1 (37/2834) |  | 1.4 (0.7~2.6) | 1.00 (ref) | 1.2 (0.7~2.2) | 1.4 (0.8~2.4) |
| BMI |  |  |  |  |  |  |  |  |  |
| Lean (<18.5) | 11.4 (5/438) | 10.0 (3/301) | 12.1 (4/331) | 37.2 (7/188) |  | 1.4 (0.3~6.0) | 1.00 (ref) | 1.0 (0.2~4.3) | 3.2 (0.8~12.6) |
| Normal (18.5–23.9) | 3.9 (15/3834) | 3.7 (14/3808) | 6.4 (21/3273) | 7.4 (20/2714) |  | 1.4 (0.7~3.0) | 1.00 (ref) | 1.6 (0.8~3.1) | 1.5 (0.7~3.0) |
| Overweight and Obesity (≥24) | 1.3 (4/2335) | 3.1 (8/2588) | 3.7 (11/2965) | 7.9 (29/3659) |  | 0.7 (0.2~2.2) | 1.00 (ref) | 1.1 (0.4~2.7) | 1.8 (0.8~4.1) |
| Obese WHR |  |  |  |  |  |  |  |  |  |
| Yes | 3.3 (11/3335) | 3.8 (13/3403) | 5.6 (20/3597) | 9.6 (38/3966) |  | 1.1 (0.5~2.6) | 1.00 (ref) | 1.3 (0.7~2.7) | 1.9 (1.0~3.5) |
| No | 4.3 (13/3056) | 3.5 (11/3129) | 5.7 (16/2810) | 6.8 (17/2485) |  | 1.6 (0.7~3.6) | 1.00 (ref) | 1.4 (0.7~3.1) | 1.5 (0.7~3.2) |
| Hypertension |  |  |  |  |  |  |  |  |  |
| Yes | 6.4 (9/1397) | 5.5 (10/1802) | 5.6 (12/2162) | 11.0 (30/2727) |  | 1.5 (0.6~3.7) | 1.00 (ref) | 0.9 (0.4~2.1) | 1.4 (0.7~3.0) |
| No | 3.0 (13/4401) | 2.7 (11/4142) | 5.9 (22/3716) | 7.3 (23/3141) |  | 1.4 (0.6~3.3) | 1.00 (ref) | 2.0 (1.0~4.2)^†^ | 2.3 (1.1~4.7)^*^ |
| Dyslipidemia |  |  |  |  |  |  |  |  |  |
| Yes | 3.6 (12/3365) | 3.8 (16/4192) | 4.1 (19/4624) | 7.4 (40/5381) |  | 1.1 (0.5~2.3) | 1.00 (ref) | 0.9 (0.5~1.8) | 1.4 (0.8~2.6) |
| No | 3.7 (12/3242) | 3.6 (9/2505) | 8.7 (17/1945) | 13.6 (16/1180) |  | 1.5 (0.6~3.7) | 1.00 (ref) | 2.2 (1.0~5.0)^†^ | 2.6 (1.1~6.0)^*^ |
| CKD |  |  |  |  |  |  |  |  |  |
| Yes | 10.5 (3/285) | 3.0 (2/676) | 15.2 (15/985) | 13.1 (20/1532) |  | 3.7 (0.6~22.0) | 1.00 (ref) | 4.9(1.1~21.7)^*^ | 4.0 (0.9~17.5) |
| No | 3.3 (21/6322) | 3.8 (23/6021) | 3.8 (21/5584) | 7.2 (36/5029) |  | 1.2 (0.7~2.2) | 1.00 (ref) | 0.9 (0.5~1.6) | 1.5 (0.9~2.5) |
| Framingham score (%) |  |  |  |  |  |  |  |  |  |
| <10 | 2.3 (12/5112) | 0.8 (3/3893) | 2.0 (6/3007) | 6.1 (11/1805) |  | 3.2 (0.9~11.4)^†^ | 1.00 (ref) | 2.6 (0.6~10.4) | 8.5 (2.3~30.7)^**^ |
| 10~20 | 5.6 (6/1070) | 7.9 (14/1781) | 6.4 (14/2188) | 4.4 (12/2710) |  | 0.7 (0.3~1.8) | 1.00 (ref) | 0.8 (0.4~1.8) | 0.6 (0.3~1.3) |
| >20 | 14.1 (6/425) | 7.8 (8/1023) | 11.6 (16/1374) | 16.1 (33/2046) |  | 1.8 (0.6~5.1) | 1.00 (ref) | 1.6 (0.7~3.6) | 2.1 (1.0~4.5) |

Abbreviation: HR, Hazards ratios; CI, confidence interval; other abbreviations are indicated in Supplementary Table 1. † P<0.10; *P < 0.05; **P < 0.01.

Supplementary Table 9. Detailed information on the diabetes

| All diabetic individuals (n=475) | |
| --- | --- |
| History of diabetes | 166 (35.0) |
| HbA1c ≥6.5% | 410 (86.3) |
| Individuals with history of diabetes (n=166) | |
| Special Diet | 79 (48.2) |
| Weight Control | 47 (28.7) |
| Oral Medcine | 129 (78.2) |
| Insulin Injection | 33 (20.1) |
| Chinese Trad Medcine | 19 (11.6) |
| Home Remedies | 9 (5.5) |

Supplementary Table 10. All-cause mortality between treatment groups among individuals with diabetes history

|  | Death | Non-death | P value |
| --- | --- | --- | --- |
| Special Diet | 3 (25.0) | 76 (50.3) | 0.091 |
| Weight Control | 3 (25.0) | 76 (50.3) | 0.337 |
| Oral Medcine | 9 (75.0) | 120 (79.0) | 0.748 |
| Insulin Injection | 4 (33.3) | 29 (19.3) | 0.247 |
| Chinese Trad Medicine | 1 (8.3) | 18 (11.9) | 0.709 |
| Home Remedies | 0 (0.0) | 9 (6.0) | 1.000 |

Data are presented as No. (%). Information of 2 individuals was missing.
